# Functional Lingual Arch with Hinge-type Lockable Dentulous Component

**DOI:** 10.5005/jp-journals-10005-1455

**Published:** 2017-02-27

**Authors:** Paul Chalakkal, Amanda N Ferreira, Godwin C Da Costa, Meena A Aras

**Affiliations:** 1 Assistant Professor, Department of Pedodontics and Preventive Dentistry, Goa Dental College & Hospital, Bambolim, Goa, India; 2 Postgraduate Student, Department of Prosthodontics & Crown and Bridge, Goa Dental College & Hospital, Bambolim, Goa, India; 3 Lecturer, Department of Prosthodontics & Crown and Bridge, Goa Dental College & Hospital, Bambolim, Goa, India; 4 Professor and Head, Department of Prosthodontics & Crown and Bridge, Goa Dental College & Hospital, Bambolim, Goa, India

**Keywords:** Functional, Hinge, Lingual arch, Molar tube, Sticker.

## Abstract

The conventional lingual arch is a nonfunctional fixed space maintainer used in the mandibular arch to maintain arch length by the prevention of mesial movement of the permanent first molars. This study highlights a functional lingual arch with a hinge-type lockable dentulous component that incorporates molar tubes, with various advantages over the conventional lingual arch.

**How to cite this article:**

Chalakkal P, Ferreira AN, Da Costa GC, Aras MA. Functional Lingual Arch with Hinge-type Lockable Dentulous Component. Int J Clin Pediatr Dent 2017;10(3):302-308.

## INTRODUCTION

Space maintainers are fixed or removable appliances used to preserve arch length, following the premature loss or elective extraction of a tooth/teeth.^1^ The lingual arch is a bilateral fixed space maintainer, consisting of a single heavy-gauge stainless steel wire adapted anteriorly to the lingual aspect of mandibular arch and posteriorly to bands on the first permanent molars.^1^ Lourie had been credited for inventing the lingual arch in 1904,^2^ although Mershon^3,4^ and Burstone are known to have popularized it.^1^ Nance, who used it extensively during the mid-1940s, indicated the lingual arch for treatment during the mixed dentition for maintenance of distance between the permanent incisors and molars.^5,6^

Arch length deficiency due to the early loss of primary teeth may result in crowding, impaction, and irregularity of the permanent dentition.^7-9^ These in turn result in loss of structural balance and functional efficiency.^10^ The greatest space loss has been attributed to the mesial movement of the permanent molars after the loss of the second primary molar.^11-13^ About 51% of first primary molars and 70% of second primary molars lost prematurely result in space loss and subsequent malposition of permanent teeth.^14^

The lingual arch prevents mesial migration of the permanent first molars,^8,15-17^ sometimes at the expense of mandibular incisor proclination.^18-21^ Reduction in arch length by the use of the lingual arch has also been reported,^22^ sometimes due to the lingual tipping of inci-sors.^15,16,20^ However, certain studies have reported that the lingual arch prevents lingual collapse of the mandibular incisors.^8,15-17^

This case report highlights a new functional lingual arch design that incorporates a hinge-type openable dentulous component with a locking mechanism, with various advantages over the conventional lingual arch design.

## CASE REPORT

**Fig. 1:**
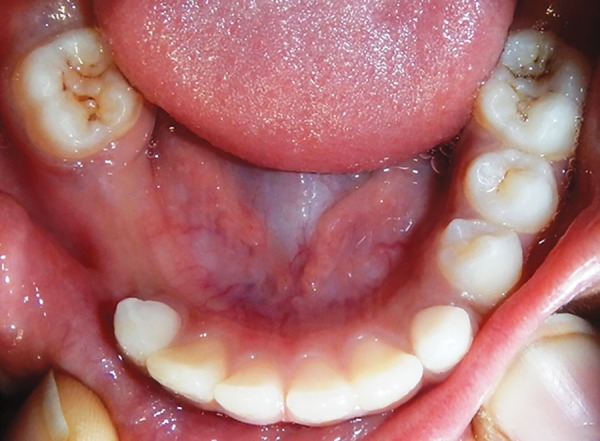
Lower arch

**Fig. 2:**
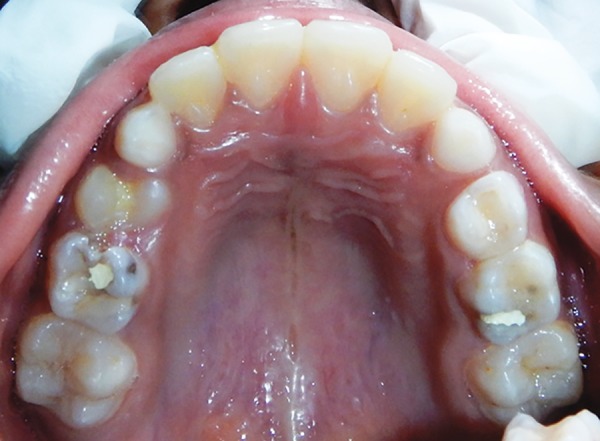
Upper arch

**Fig. 3:**
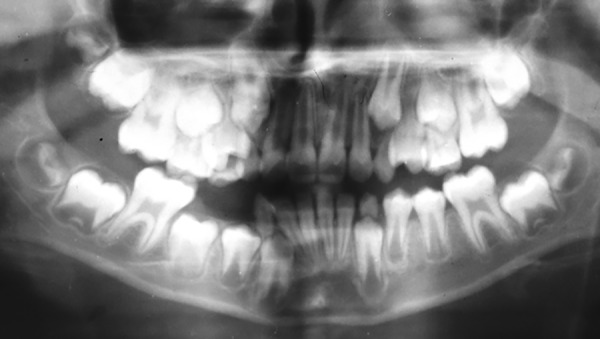
Orthopantomograph

**Fig. 4:**
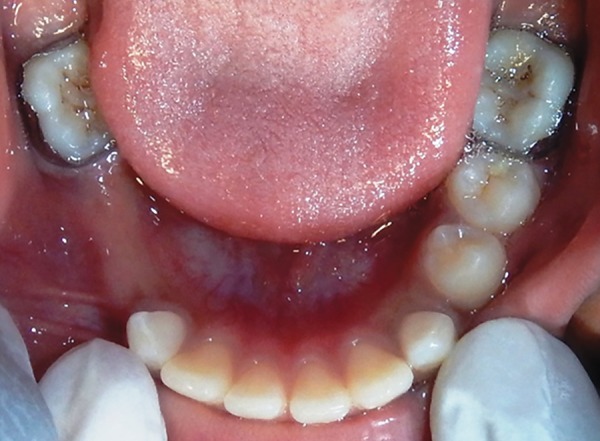
Banded 36 and 46

**Fig. 5:**
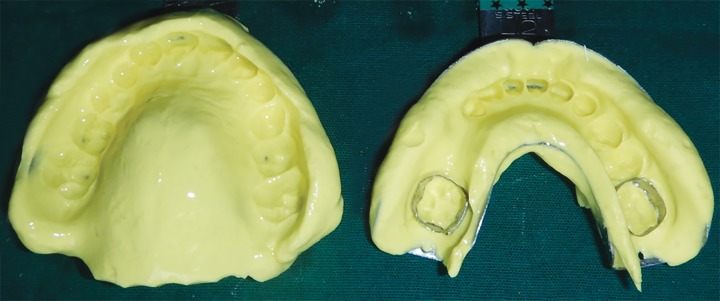
Alginate impressions after placement of bands

**Fig. 6:**
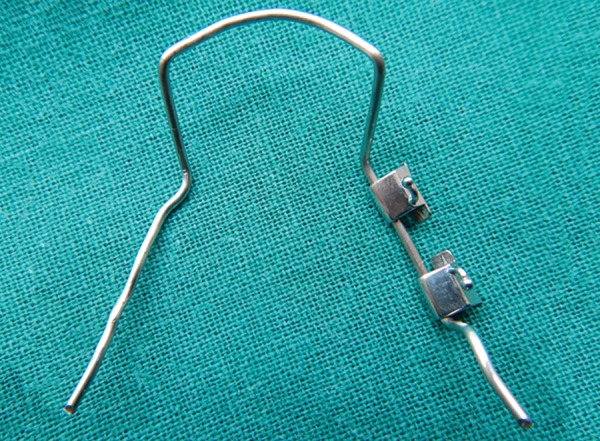
Lingual arch incorporating molar tubes

**Fig. 7:**
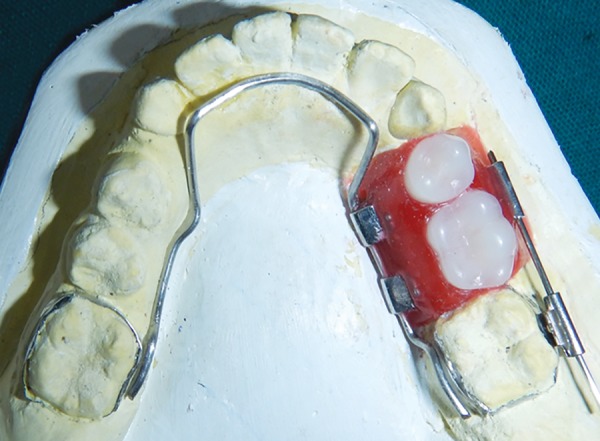
Lingual arch with wax up

**Fig. 8:**
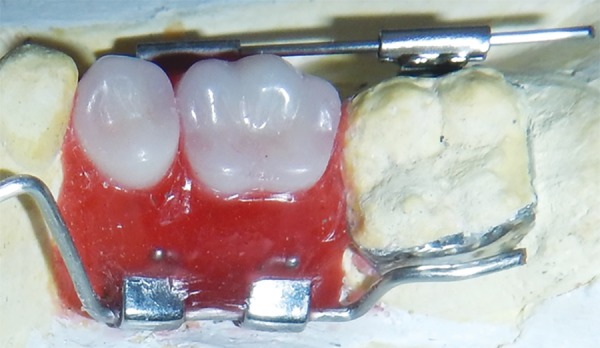
Wax up (lingual view)

**Fig. 9:**
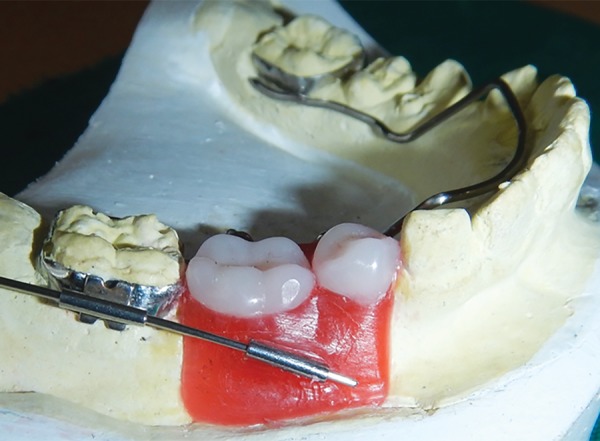
Wax up (buccal view)

**Fig. 10:**
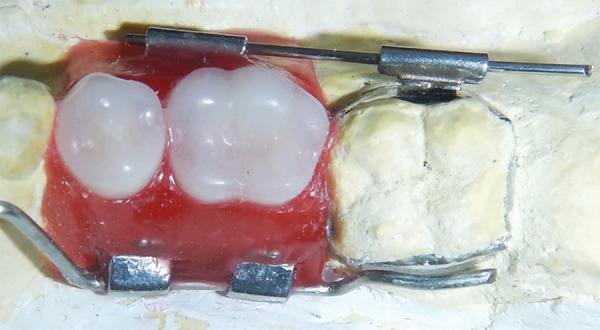
Wax up (superior view)

**Fig. 11:**
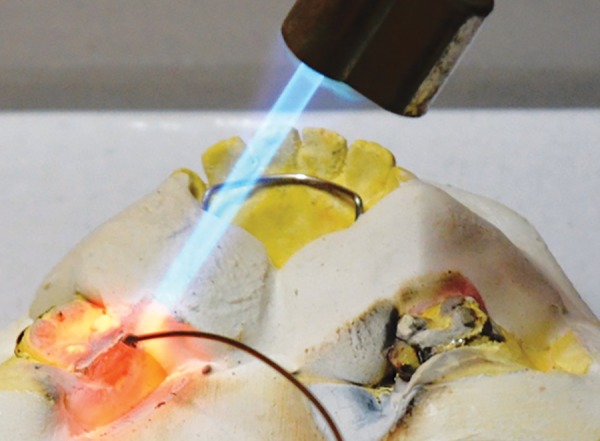
Soldering

A 10-year-old boy visited the Department of Pedodontics with the complaint of being unable to chew on his right side. Upon examination, the lower arch contained the following teeth: 36, 35, 34, 73, 32, 31, 41, 42, 83, and 46 (Fig. 1). The upper arch contained teeth consistent with the intertransitional period, except that 14 had erupted (Fig. 2). Upon history taking, it was gathered that 84 and 85 had been extracted a year ago due to extensive caries and severe pain. An orthopantomograph (OPG) revealed half root completion of the unerupted 44 and 45 (Fig. 3). It was decided to construct a LHLD (functional lingual arch with hinge-type lockable dentulous component) in order to maintain the edentulous span until the eruption of 44 and 45, and to relieve the child of his chewing disability. After band adaptation on 36 and 46, alginate impressions were made of both the arches, into which the prepared bands were placed (Figs 4 and 5). Casts were poured with dental stone. A lingual arch incorporated with two molar tubes (on the edentulous side) was fabricated using 0.9 mm wire on the lower cast (Fig. 6). Wax up was done on the edentulous span containing artificial molar teeth (to replace 84 and 85), such that the molar tubes were incorporated into the wax up in order to later serve as hinges for the dentulous component. On the buccal side of the dentulous component, another molar tube was placed, such that it was parallel and in alignment with another buccal tube welded on the buccal surface of the molar band (Figs 7 to 10). The lingual arch was then soldered to the bands (Fig. 11).

**Fig. 12:**
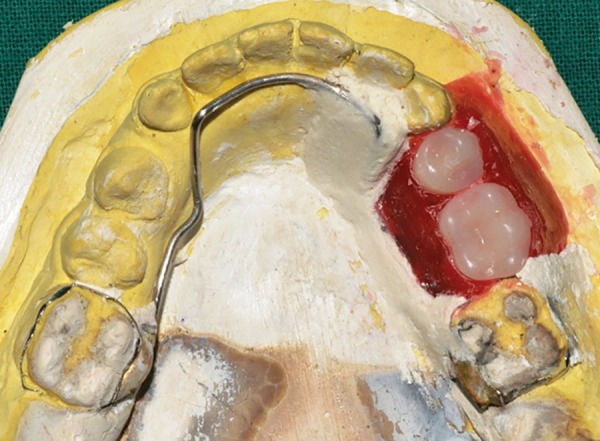
Plaster blockout of wax up

**Fig. 13:**
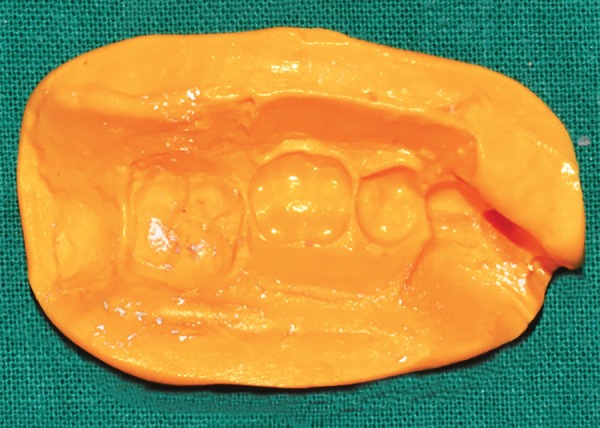
Index of wax up

**Fig. 14:**
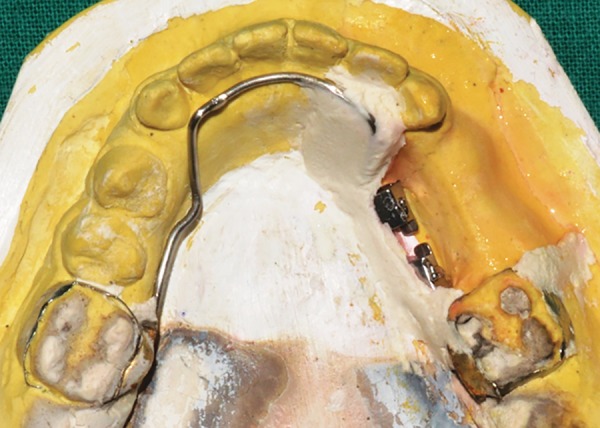
After dewaxing

**Fig. 15:**
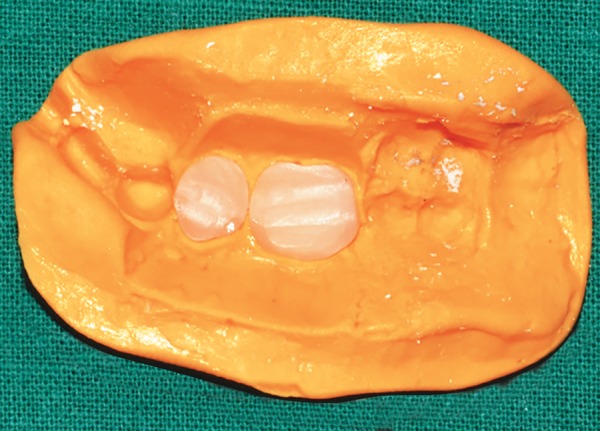
Artificial teeth placed in the index

**Fig. 16:**
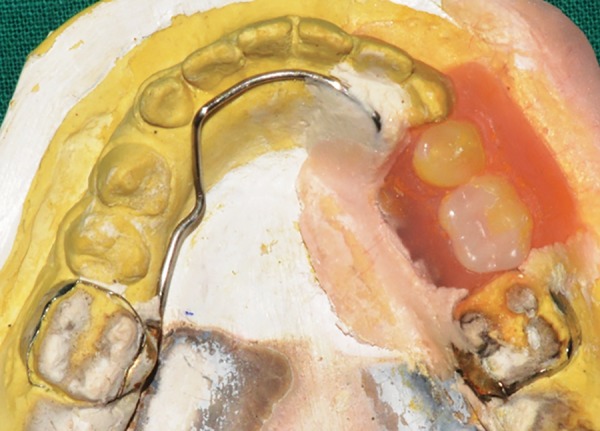
After acrylization

**Fig. 17:**
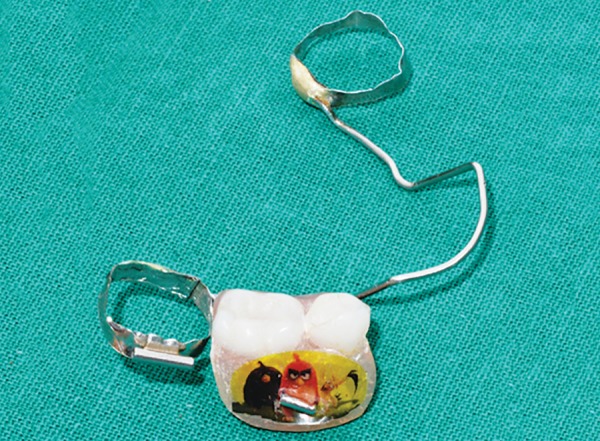
Appliance (buccal view)

**Fig. 18:**
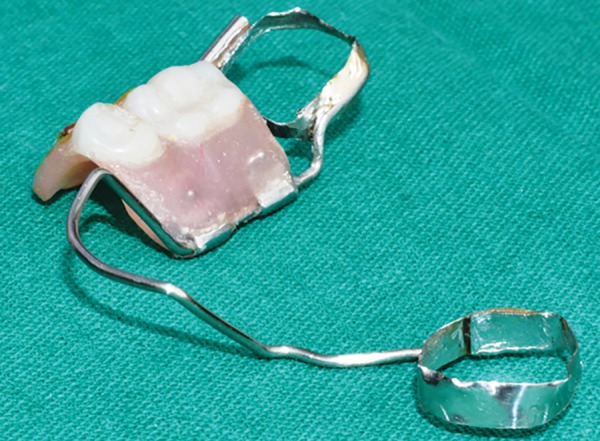
Appliance (lingual view)

Plaster blockout of the undercuts and buccal tubes was done (Fig. 12). Acrylization was carried out using self-cure acrylic by the putty method. An index was made of the wax up (Fig. 13) using addition silicone (Elite HD, Zhermack, Germany), following which the cast was dewaxed (Fig. 14). Artificial teeth were incorporated into the index (Fig. 15) and self-cure acrylic was mixed with monomer and placed into the index. The index was reseated on the edentulous ridge on the cast until the acrylic mixture had completely polymerized (Fig. 16). An "Angry Birds" sticker was laminated and placed on the buccal surface of the dentulous component and covered with a thin layer of clear acrylic. The appliance was then gently removed from the cast (Figs 17 and 18).

**Fig. 19:**
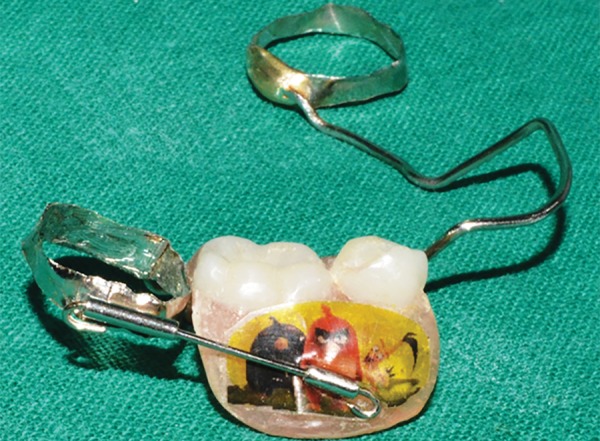
Appliance (with wire passed into locking component)

**Fig. 20:**
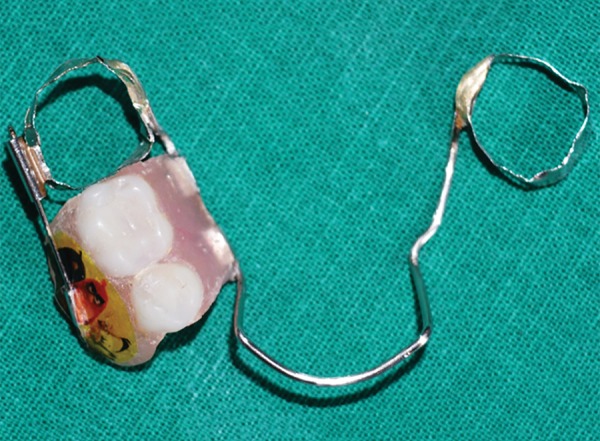
Appliance (superior view)

**Fig. 21:**
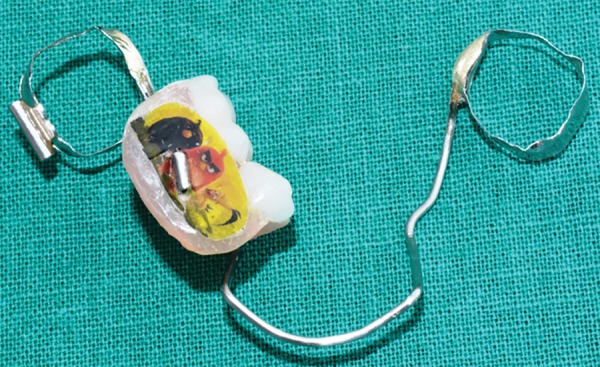
Appliance (with dentulous fragment opened)

**Fig. 22:**
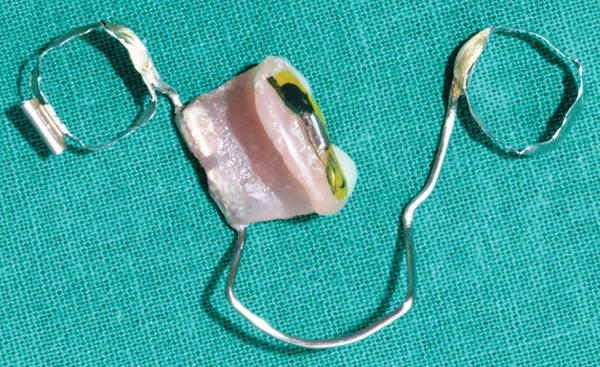
Appliance (with dentulous fragment opened)

**Fig. 23:**
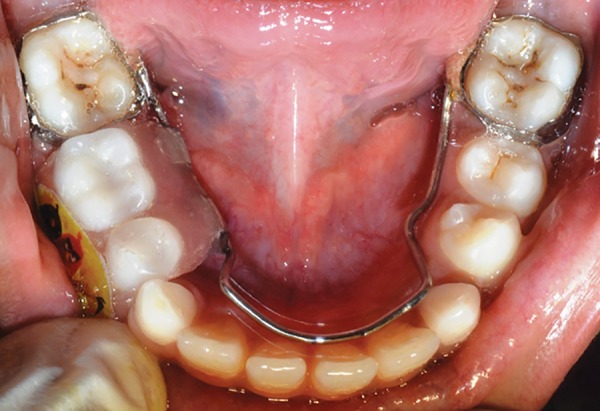
Appliance (intraoral occlusal view)

**Fig. 24:**
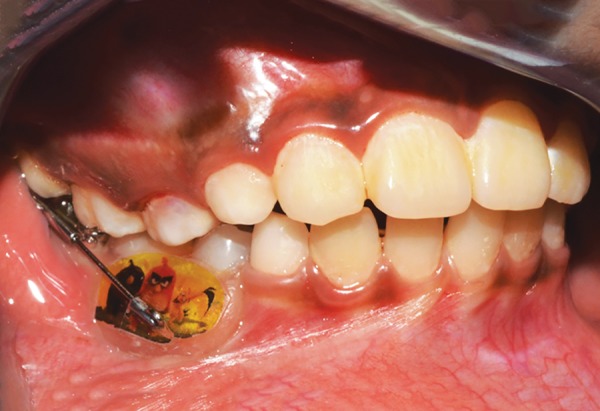
Appliance (intraoral buccal view)

The tubes on the buccal surface were incorporated in order to pass the locking wire (0.64 mm) that formed the locking component of the appliance (Figs 19 and 20). The efficacy of the hinge (molar tubes attached to the lingual arch) was verified by rotating the dentulous component around its hinge axis (Figs 21 and 22). Finishing and polishing was carried out for the acrylic and metal components. The appliance was evaluated for any mucosal interferences or occlusal disharmonies, following which the appliance was inserted intraorally by cementing the bands onto 36 and 46 using luting glass-ionomer cement (Figs 23 and 24). The patient was recalled the next day for a check-up, following which another general evaluation was done after a week.

## DISCUSSION

Leeway space represents the difference between the sum of the mesiodistal diameter of the primary canine and molars and the sum of the mesiodistal diameter of the permanent canine and premolars^22^ and can measure up to 4.3 mm.^23^ As much as 4.8 mm of space becomes available after the permanent canines and premolars replace their primary successors.^24^ Normally, the first molars move mesially into the leeway space, decreasing the arch length.^23^ This space can be preserved by maintaining the arch length with passive appliances, such as the lingual arch.^8/15/16/21/22/25/26^ In the current case, although extraction of 84 and 85 was carried out a year ago, there was no resultant loss in arch length when the distances between permanent molars and primary canines were measured on both the mandibular quadrants and compared. Moreover, space analysis had shown the presence of sufficient space for the eruption of 44 and 45. There was no loss in arch length probably because the extraction of 84 and 85 were carried out during the second transitional period, rather than the first or intertransitional periods.

Early loss of primary molar teeth can eventually cause narrowing in the dental arch.^13^ Although the extraction of primary molars had been carried out a year ago, narrowing of the corresponding region of the arch was observable.

Space maintenance is indicated when the succeeding tooth has < 75% of root formation, or when there is >1 mm of alveolar bone above it.^7^ Such was the situation in the patient as per the OPG with regard to 44 and 45. The thickness of wire used for the construction of the LHLD was 0.9 mm. Lingual arches made with 0.9 mm stainless steel wire are superior in terms of arch length preservation.^18^ Owais et al^18^ showed that 1.25 mm wire increased the stiffness, resulting in increased forces on the lower incisors and first molars, as compared with 0.9 mm wire. This resulted in greater proclination of the incisors and loss of the Leeway space. Additionally, increased wire stiffness resulted in higher cementation failure and wire/band breakage.

A conventional lingual arch has the following drawbacks: (1) Risk of extrusion of antagonist teeth into the lower edentulous space and (2) nonfunctional (does not bear artificial teeth).^27^ However, the LHLD does not have any of the above disadvantages. Never before in dental literature has a similar appliance been fabricated. It has the following advantages:

- The hinge-type design provides easy visualization of the ridge by opening the dentulous component around its hinge axis without having to deband the lingual arch, after the locking component is opened. Periodic inspection of the ridge is required to check for any mucosal alterations and to check for eruption of the premolars. The locking component can be opened by cutting and removing the wire that passes through the molar tubes on the buccal aspect. After inspection, a new wire can be passed through the tubes and turned into a loop mesially and distally, in order to lock the dentulous component.
- The appliance helps in mastication since it is functional.
- The underside of the dentulous component can be cleaned upon opening the dentulous component.
- There is prevention of overeruption of antagonist teeth

It also satisfies all the requirements of an ideal space maintainer, such as: (1) preservation of space; (2) allows for eruption of adjacent, succedaneous, and abutment teeth; (3) restoration of masticatory function; (4) prevention of over eruption of antagonist teeth; (5) compatibility with soft tissues; (6) effective hindrance of torquing forces on abutment teeth; (7) economy of construction and resistance to distortion; (8) allowance for adjustment or minor repair; and (9) universal application.^28^

However, since it is a fixed appliance with an acrylic component, maintenance of oral hygiene would often be difficult. This remains the disadvantage of the appliance.

## CONCLUSION

The LHLD has various advantages over the conventional lingual arch for being a functional space maintainer for use in the lower arch.
